# A Case of Steroid-Responsive Acute Tubular Injury of Unknown Trigger: A Case Report

**DOI:** 10.7759/cureus.52304

**Published:** 2024-01-15

**Authors:** Ali Alqaraishi, Mohammed Tawhari, Nawaf M Alyahya, Alanoud Alotaibi, Alanoud Ghoulah, Abdulrahman Aledrees, Abdulrahman Alabdulsalam

**Affiliations:** 1 Internal Medicine, King Abdulaziz Medical City, Ministry of National Guard Health Affairs, Research Center, King Abdullah International Medical Research Centre, Riyadh, SAU; 2 Nephrology, King Abdulaziz Medical City, Riyadh, SAU; 3 Nephrology, King Abdullah International Medical Research Centre, Riyadh, SAU; 4 Nephrology, King Saud Bin Abdulaziz University for Health Sciences College of Medicine, Riyadh, SAU; 5 Internal Medicine, College of Medicine, Imam Abdulrahman Bin Faisal University, Khobar, SAU; 6 Internal Medicine, College of Medicine, King Saud Bin Abdulaziz University for Health Sciences, Riyadh, SAU; 7 Pathology and Laboratory Medicine, King Abdulaziz Medical City, Riyadh, SAU

**Keywords:** kidney biopsy, role of steroids, pulse steroids, acute kidney injury care, acute tubular necrosis (atn)

## Abstract

Acute tubular necrosis (ATN) is a serious medical condition characterized by the rapid destruction of renal tubular epithelial cells, resulting in acute kidney injury, given its multifactorial etiologies, which can include nephrotoxic agents, ischemic insults, hypovolemia, and sepsis. We report the case of a young male patient who presented with recurrent worsening kidney function with bland sediment that was confirmed with multiple kidney biopsies as recurrent attacks of ATN of unclear etiology, which did not respond to supportive measures but did respond to steroids.

## Introduction

Acute tubular necrosis (ATN) is a serious medical condition characterized by the rapid destruction of renal tubular epithelial cells, resulting in acute kidney injury (AKI). Given its multifactorial etiologies, which can include nephrotoxic agents, ischemic insults, hypovolemia, and sepsis [1], it remains a substantial challenge in clinical practice to determine the specific etiology of ATN [1]. ATN is a frequent cause of AKI in hospitalized critically ill patients, and its timely identification and management are crucial in preventing complications and improving prognosis [2].

The clinical manifestations of ATN are diverse, with patients frequently presenting with nonspecific symptoms such as malaise, fatigue, fluid overload, and elevated serum creatinine levels, indicating impaired renal function [3]. Furthermore, the severity of the condition is emphasized by the existence of other laboratory findings, such as metabolic disorders and electrolyte imbalance [3].

The pathophysiology underlying ATN involves a complicated interplay of numerous mechanisms [4]. Ischemic ATN is usually caused by conditions such as sepsis, congestive heart failure, and severe dehydration. The condition occurs due to decreased renal perfusion, leading to insufficient delivery of oxygen and nutrients to the renal tubules. On the other hand, nephrotoxic ATN is the outcome of the direct toxic effects of certain agents, such as aminoglycoside antibiotics, iodinated contrast media, and nonsteroidal anti-inflammatory drugs, on the renal tubular cells [5]. These insults activate a cascade of events that include cellular injury, inflammation, oxidative stress, and apoptosis, culminating in tubular necrosis [4,5].

The cornerstone of treatment for ATN revolves around addressing the underlying cause and providing supportive care to address the complications [6]. Management involves administering intravenous fluids to maintain adequate intravascular volume, correction of electrolyte imbalance, and avoidance of nephrotoxic medications when feasible [6]. Furthermore, attentive and careful monitoring of the patient’s fluid and electrolyte status is essential to prevent further complications [7].

In this case report, we present the case of a patient diagnosed with repeated attacks of ATN with an unclear trigger, not responding to supportive measures. However, he experienced a successful response to a carefully tailored corticosteroid regimen. This case report aims to shed light on the potential benefits and obstacles associated with steroid therapy in ATN, considering its underlying pathophysiology and variable patient characteristics.

## Case presentation

A previously healthy 21-year-old male presented to the emergency department complaining of three episodes of food content vomiting, non-projectile, nonbloody, associated with dizziness, fatigue, and decreased oral intake. He denied any constitutional or systemic symptoms and any use of medications. Upon admission, he was alert and oriented, afebrile, had a blood pressure of 163/91 mmHg, and his heart and respiratory rates were within the normal range. Although his physical examination revealed no significant findings, only mild dehydration was noted, likely due to vomiting episodes.

The patient underwent basic labs, including a complete blood count, kidney function profile, electrolytes, and urinalysis (Table 1). As the urine protein-to-creatinine ratio and albumin-to-creatinine ratio were unremarkable, the patient was admitted to the nephrology department for eight days to investigate potential renal causes of AKI. During this period, a comprehensive workup for glomerulonephritis was conducted. Renal ultrasound demonstrated normal kidney size and echogenicity without hydronephrosis. Serological investigations for antinuclear antibodies, anti-double-stranded DNA, myeloperoxidase-antineutrophil cytoplasmic antibody, proteinase 3-antineutrophil cytoplasmic antibody, C3 and C4 complement levels (Table 2), and anti-hepatitis B surface antigen was non-reactive. Additional screenings for porphyria, creatine phosphokinase, urine toxicology, angiotensin-converting enzyme, serum protein electrophoresis, and free light chain produced negative findings. Immunoglobulin G levels and its subclasses were also within normal limits.

**Table 1:** Laboratory findings at all admissions.

	Laboratory findings				
	Reference range	First admission	Second admission	Third admission	Fourth admission
Urinalysis blood	<2 red blood cells	0.10	0.10	0.10	0.50
Urinalysis protein	Negative, mg/dL	10	100	100	100
Urinalysis red blood cells	0–5/HPF	16	91	76	43
Urinalysis hyaline casts	None/LPF	0-2	-	-	-
Creatinine	64–110 µmol/L	931	170	181	300
Blood urea nitrogen	3.2–7.4 mmol/L	24.7	7.7	8.7	9.8
Estimated glomerular filtration rate	<116 ml/minute/1.73m^2^	7	47	43	24
Uric acid	210–420 µmol/L	923	1,188	1,103	1,461
Phosphorous	1.12–1.45 mmol/L	1.90	2.12	2.56	2.06
Calcium	2.2–2.7 mmol/L	2.25	2.40	2.18	2.16
Potassium	3.5–5.2 mEq/L	3.9	4.7	4.5	4.5
Albumin	35–55 g/L	41	49	43	44
Magnesium	0.85–1.20 mmol/L	1.33	1.45	1.52	1.70
Sodium	135–145 mmol/L	136	139	140	137
Chloride	96–106 mmol/L	100	106	106	102
Hemoglobin	13.8–17.2 g/dL	142	140	142	163
Red blood cell	4.7–6.1 million cells/µL	4.33	4.21	4.89	4.02
White blood cell	4,500–11,000 WBCs/µL	10.20	14.90	15.90	18.90
Platelet	150,000–400,000 platelets/µL	254	255	261	242

**Table 2:** Serology findings.

Serology at the first admission	
Antinuclear antibodies (<1.0 IU)	8.90
Anti-double-stranded DNA (<200 IU/mL)	20.94
Myeloperoxidase-antineutrophil cytoplasmic antibody (<3.5 IU/mL)	1.38
Complement C3 (0.88–2.01 g/L)	1.370
Complement C4 (0.15–0.45 g/L)	0.256
Proteinase 3-antineutrophil cytoplasmic antibody (<2 IU/mL)	2.82

**Figure 1:**
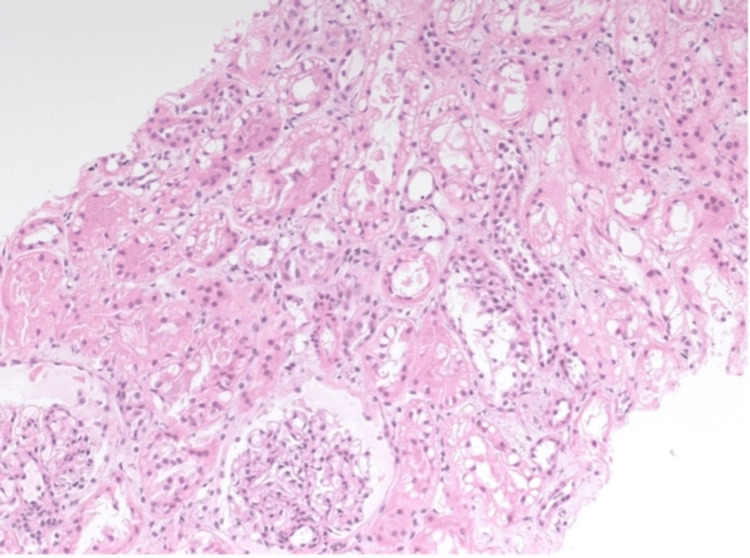
Core from the renal cortex showing mild interstitial edema and diffuse tubular injury, mostly manifesting as cytoplasmic sub-nuclear vacuoles (hematoxylin and eosin stain, 200× and 400×, respectively).

**Figure 2:**
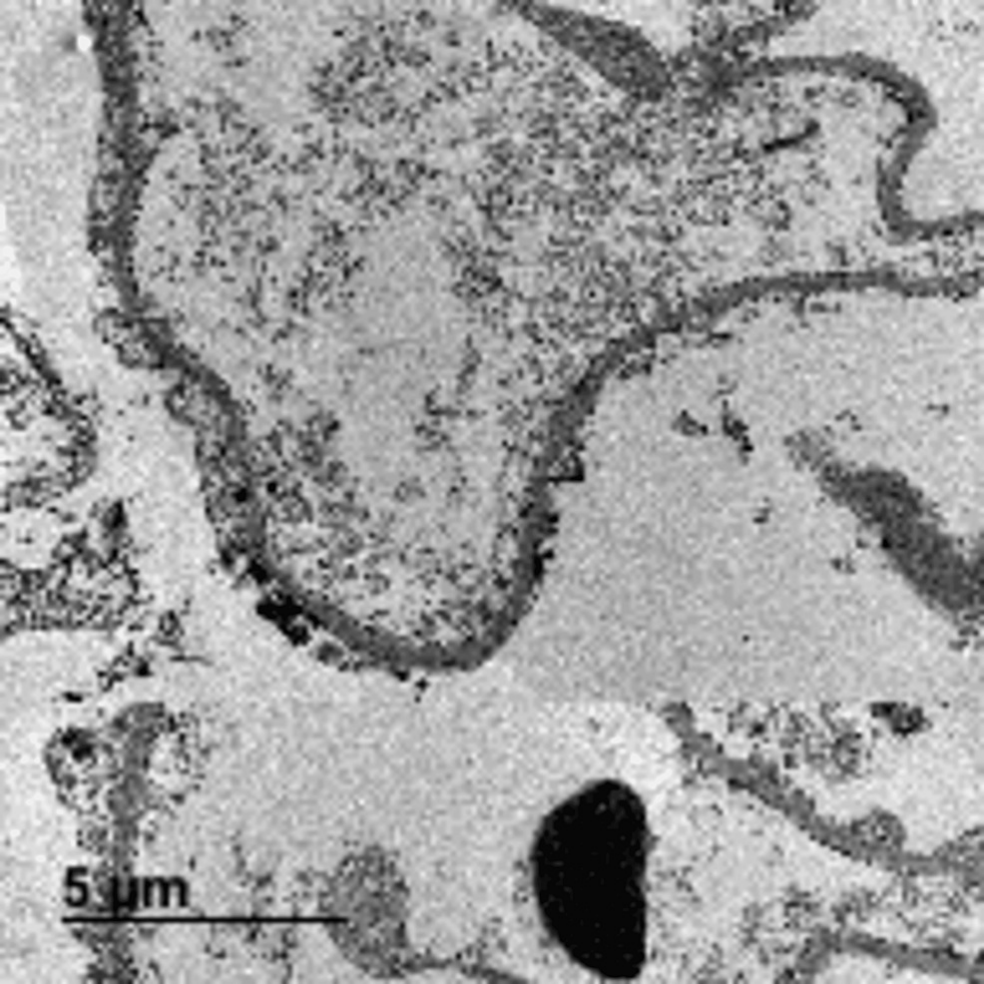
Ultrastructural examination shows effacement of the podocyte foot process, while the glomerular basement membrane has unremarkable thickness and texture (EM).

Considering the severity of AKI and given that the patient presented during holidays, the treating physician decided to initiate the patient on pulse-steroid therapy while awaiting a kidney biopsy. The patient initially received a three-day course of methylprednisolone through intravenous (IV) injection at a dose of 500 mg per day, which was then switched to oral steroids, prednisolone, for two days at a dosage of 60 mg per day. After the treatment period, kidney function gradually improved over a week, from 931 μmol/L to 100 μmol/L. Finally, a kidney biopsy was performed, which showed diffuse tubular vacuolization and focal interstitial edema (Figure 1), while glomeruli only showed evidence of podocyte injury (foot process effacement over 60% of the capillary surface) (Figure 2), possibly suggesting early interstitial nephritis. Given the biopsy results of ATN, the nephrology team decided to stop the steroid therapy, and the patient was discharged and was seen at the clinic a week later when his kidney function and urinalysis were done. His kidney function profile improved to a normal level and his urinalysis was completely unremarkable.

One month later, the patient was readmitted to the nephrology department with a recurrent complaint of multiple episodes of vomiting, accompanied by fatigue and lethargy, with no other associated symptoms. The patient was conscious, alert, and oriented, with a blood pressure of 143/72, and was otherwise vitally stable. His laboratory investigations revealed a deranged kidney function profile and leukocytosis. His urinalysis showed microscopic hematuria and proteinuria (Table 1), with no signs of infection. Additionally, considering the previous event and the response to steroids, treatment commenced with IV methylprednisolone at a dosage of 500 mg for three days. The patient’s creatinine level improved to 114 μmol/L, and he was subsequently discharged on oral prednisolone at a dose of 60 mg twice daily with a tapering dose of 5 mg weekly. To ensure patient adherence to steroids, he was seen in the nephrology clinic weekly and follow-up clinic appointments were scheduled to monitor his kidney function and consider a tapering dose of prednisolone. As the patient improved on steroids, the treating team decided not to repeat the kidney biopsy.

**Figure 3:**
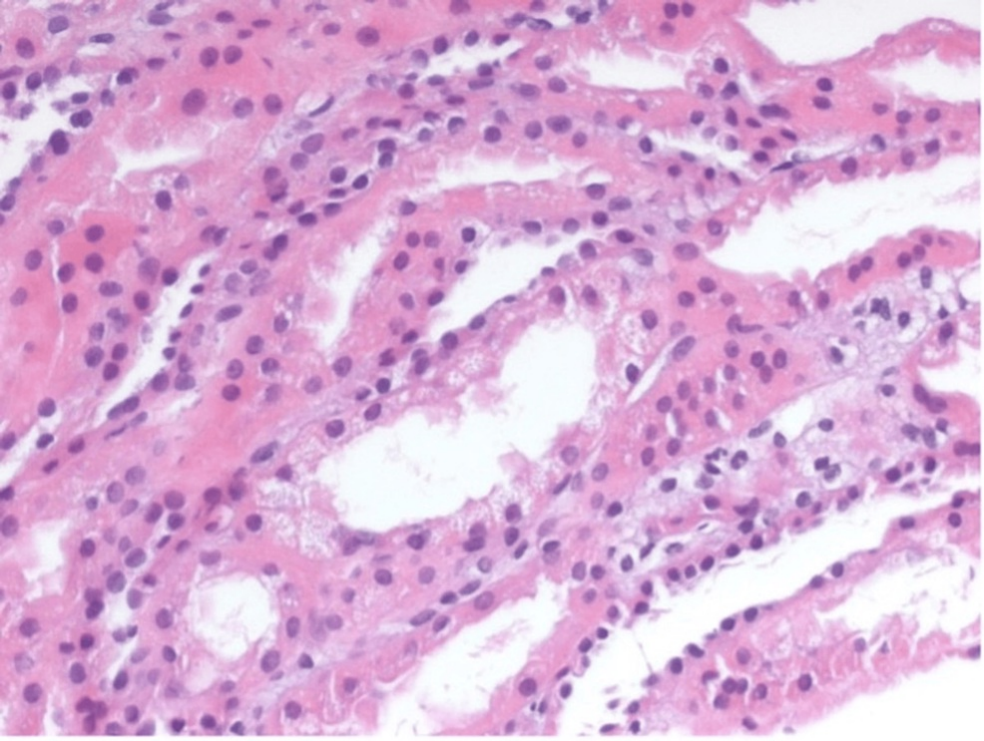
Tubular vacuolization and focal interstitial lymphocytic infiltrate (hematoxylin and eosin stain, 400×).

One week after discontinuation of prednisolone, he presented with a similar complaint of vomiting persisting for three days and no other systemic symptoms. On presentation, it was discussed that the first kidney biopsy results may have been affected by the steroids, so the team decided to conduct a second biopsy before administering any steroids. During the admission, IV fluids were given. The creatinine level was 181 μmol/L and blood urea nitrogen was 8.7 mmol/L (Table 1). While awaiting the biopsy results, methylprednisolone was introduced at a dosage of 250 mg for a three-day course, resulting in an improvement in his creatinine levels, which decreased to 100 μmol/L. The biopsy showed a similar but less profound tubular injury and focal interstitial lymphocytic infiltrate (Figure 3). In both biopsies, we could not appreciate any significant immunofluorescence staining. Moreover, the biopsy was examined under a polarized microscope and was negative for any crystals. Overall, the findings were consistent with acute tubular injury. After the IV steroids, his kidney function enzymes normalized, and he was discharged on prednisolone 60 mg, which was gradually tapered down to 10 mg daily.

Following days after the discharge, once the patient was on 10 mg of prednisolone, he was readmitted for the fourth time with complaints of vomiting for a few days. IV fluids were administered, and a pulse-steroid regimen of methylprednisolone 500 mg was initiated for three days with significant clinical and biochemical improvement. Given the repetitive courses of steroids and considering their long-term side effects, the patient was initiated on mycophenolate mofetil 1,000 mg orally twice daily along with prednisone 30 mg with tapering down the dose to 5 mg/day. Since then, the patient has been seen in the clinic a few times with a repeat analysis of creatinine levels at each visit (Table 3).

**Table 3:** Follow-up creatinine levels in the clinic.

Creatinine levels	One month	Three months	Six months
64–110 μmol/L	92 μmol/L	74 μmol/L	73 μmol/L

## Discussion

We report a case of acute renal failure due to ATN with no clear trigger. ATN is one of the most common causes of AKI, especially in hospitalized patients, with the mortality rate reaching up to 80% [8]. It can be caused by multiple factors, mostly ischemia and sepsis [8].

Fluid therapy is widely recognized as the prevailing method for managing ATN [2]. Its primary objectives include preserving intravascular volume, enhancing renal perfusion, and preventing hypotension and nephrotoxin exposure to mitigate the risk of additional kidney damage [2].

Multiple pharmacologic agents have been studied for the treatment of ischemic ATN. It is important to note that diuretics are not used as a treatment for ATN, although they may be used to manage volume status. This is consistent with the 2012 Kidney Disease Improving Global Outcomes guidelines [9]. Studies have shown that although diuretics can increase urine output, they do not have any effect on kidney function or survival among patients with ATN [10,11].

In our case, we believed that the patient did not exhibit signs of severe volume depletion, which would make pre-renal causes of his AKI less likely, and the fact that the patient did not respond to fluid therapy also supports this observation.

It is interesting to note that in recent years, there has been increased attention toward the use of corticosteroids in the treatment of ATN, especially in cases where there are suspected inflammatory components contributing to tubular injury [9]. Corticosteroids are known for their immunomodulatory effects and may help mitigate tubular damage by reducing the inflammatory response in the renal tubules [12]. However, there are no established studies or case reports indicating the role of steroids and immunosuppressive therapy in ATN.

Despite this, the patient’s improvement following steroid therapy and mycophenolate mofetil administration in this case raises questions about the potential benefits of this approach. This is particularly noteworthy as there were repetitive attacks of ATN whenever the steroid dose was tapered down, and there was no clear trigger for the underlying ATN. It may be worthwhile to conduct further clinical trials and research on the topic of steroids and immunosuppressive therapy to explore their potential role in the management of ATN. Additionally, it is worth noting that, although rare, there are cases where patients may not respond to fluid resuscitation, which again highlights the importance of exploring different ways or approaches to ATN treatment.

## Conclusions

ATN is a common cause of AKI. While fluid therapy is often used as the main treatment, some patients may not respond adequately to fluid resuscitation alone. In some cases of ATN where no trigger can be identified, the initiation of steroids followed by steroid-sparing agents may be helpful. However, it is important to keep in mind that further evidence, studies, and randomized trials are needed to fully understand the potential benefits of this treatment approach for immune-mediated ATN.
